# *In Vitro* Cadmium-Induced Alterations in Growth and Oxidative Metabolism of Upland Cotton (*Gossypium hirsutum* L.)

**DOI:** 10.1155/2014/309409

**Published:** 2014-06-11

**Authors:** M. K. Daud, Lei Mei, Ullah Najeeb, Muhammad Azim Khan, Farah Deeba, Irum Raza, Aliya Batool, S. J. Zhu

**Affiliations:** ^1^Department of Agronomy, College of Agriculture and Biotechnology, Zhejiang University, Zijingang Campus, Hangzhou 310058, China; ^2^Department of Biotechnology and Genetic Engineering, Kohat University of Science and Technology, Kohat 26000, Pakistan; ^3^Department of Weed Science, The University of Agriculture, Peshawar, Pakistan

## Abstract

Cadmium (Cd) is a toxic pollutant, which cause both dose- and time-dependent physiological and biochemical alterations in plants. The present *in vitro* study was undertaken to explore Cd-induced physiological and biochemical changes in cotton callus culture at 0, 550, 700, 850, and 1000 **μ**M Cd for four different stress periods (7, 14, 21, and 28 days). At 1000 **μ**M Cd, mean growth values were lower than their respective control. The cell protein contents decreased only after 7-day and 14-day stress treatment. At 550 **μ**M Cd, malondialdehyde (MDA) contents decreased after various stress periods except 21-day period. Superoxide dismutase (SOD) activity at 1000 **μ**M Cd improved relative to its respective controls in the first three stress regimes. Almost a decreasing trend in the hydrogen peroxide (H_2_O_2_) and peroxidase (POD) activities at all Cd levels after different stress periods was noticed. Ascorbate peroxidase (APX) activity descended over its relevant controls in the first three stress regimes except at 700 **μ**M Cd after 14- and 21-day stress duration. Moreover, catalase (CAT) mean values significantly increased as a whole. From this experiment, it can be concluded that lipid peroxidation as well as reactive oxygen species (ROS) production was relatively higher as has been revealed by higher MDA contents and greater SOD, CAT activities.

## 1. Introduction

Cadmium (Cd) is a significant environmental pollutant due to having high toxicity and large solubility in water [1]. Cd has close chemical and physical similarities to cations (Fe, Cu, and Zn) and thus can easily enter the food chain and causes numerous health problems such as cancer. Unlike Fe, Cu, and Zn, it is a nonredox metal with strong phytotoxic [2] nature. Cd induces various functional-based alterations in plants. They are such as growth retardation, chlorosis and necrosis of leaves, red-brown coloration of leaf margins or veins, changes in root morphology, root and leaf anatomy, and damages to cell structures as well as disturbance in water balance, mineral nutrition, photosynthesis, respiration, and plant development [3].

Cd can cause oxidative damage by stimulating the free radical production [4–7] in the form of reactive oxygen species (ROS). ROS such as hydrogen peroxide (H_2_O_2_), superoxide radical (O_2_^−^), and hydroxyl radical (OH^−^) can alter membranes' function by changing lipid composition [8–10] as well as affecting the enzymatic activities, for example, H + ATPase associated with membranes [11]. Against such oxidative damage, plants activate various antioxidative enzymes system, namely, SOD, POD, APX, and CAT. They are the most important components in the scavenging system of ROS [12]. SOD is the major O_2_^−^ scavenger, which produces H_2_O_2_ and O_2_ as a result of its enzymatic action. H_2_O_2_ is broken down into H_2_O and O_2_ by the action of CAT and several classes of peroxidases [13]. APX presents the ascorbate-dependent H_2_O_2_-scavenging mechanism in plants, which reduces H_2_O_2_ to H_2_O using ascorbic acid as an electron acceptor. MDA formation is a general indicator of peroxidation of lipids [14, 15] in the membranous bodies of cell.

*In vitro* culture can rightly provide uniformly controlled environmental conditions in order to study various physiological and biological processes in plants. It provides better opportunity to develop new germplasm according to the changing demands [16–19]. Callus cultures of plant species such as tobacco, sunflower, soybean, coffee, and sugar cane have been extensively used to understand the mechanism of metal resistance [20, 21].* In vitro *culturing of plant cells' in the presence of high concentrations of metals provides a useful tool to better comprehend the adaptive mechanisms of plants living in adverse environments. Although efficient antioxidant system in plants is undoubtedly involved to combat heavy metal stress, the variations in the degree of responses have demonstrated that multiple mechanisms rather than a single mechanism may be responsible for the adaptation of the tissues to resist metal stress [2].

Cotton has been one of the first plant species used for callus induction and somatic embryogenesis studies. It has been previously studied for salinity stress both at the callus [22, 23] and at the whole plant level [12]. However, not a single study has been undertaken in cotton callus culture regarding the heavy metal toxicities particularly Cd. Keeping in view the global importance of cotton in* in vitro* studies, toxic nature of the Cd, and lack of information regarding the cellular responses of cotton callus against Cd stress, the present experiment was undertaken. The main objective was to evaluate functional and oxidative alterations in cotton callus under Cd stress.

## 2. Materials and Methods

### 2.1. Growth of Callus Culture

Matured uniform-sized seeds of upland cotton (cv. YZ1) were decoated. Coatless seeds were surface sterilized by 70% (v/v) ethyl alcohol for 3 minutes, followed by 0.1% (w/v) HgCl_2_ for 8 minutes. They were then germinated on MS (Murashige and Skoog) basal medium [24] supplemented with 1.5% (w/v) glucose and 0.25% (w/v) phytagel at 28 ± 2°C in the dark for 3 days. The germinated seedlings were transferred to the culture room (28 ± 2°C) under a 14 : 10 day : night photoperiod for 7 days. Then, 3-4 mm cuttings of hypocotyls of the seedlings were transferred to MSB_5_ (MS + Gambourg B_5_) callus induction medium by adding 0.5 mg/L 2, 4-D, 0.15 mg/L KT, 3% (w/v) glucose, and 0.25% (w/v) phytagel. Induced calli were subcultured on fresh MSB_5_ callus induction medium to get nonembryogenic callus. After three months of subculturing, well-proliferated nonembryogenic calli were transferred to MSB_5_ embryogenic callus induction medium supplemented with 0.5 mg/L IBA, 0.15 mg/L KT, 1 g/L glutamine, 0.5 g/L asparagines, 3% (w/v) glucose, and 0.25% (w/v) phytagel. The parrot green color embryogenic calli were successfully obtained after subculturing for 3-4 times (about 3 months). Moreover, pH 5.8 in different media was maintained by adding 0.1 N NaOH or HCl and each subculturing was performed after 3-4 weeks. After 8 months, embryogenic callus with high proliferation rate was obtained, which was used to study the Cd stress related physiological and biochemical changes.

### 2.2. Supplementation of Cd Stress

In order to study Cd stress in the embryogenic callus culture of upland cotton, five different levels of Cd in *μ*M, that is, 0, 550, 700, 850, and 1000, were applied. Light parrot green embryogenic calli with high proliferation rate were used. Both the stresses were singularly applied in the MSB_5_ embryogenic callus induction and proliferating medium before autoclaving and pH was adjusted to 5.8. Data were taken for four different stress periods with one week interval, that is, 7, 14, 21, and 28 days.

### 2.3. Determination of Relative Fresh Weight and Percent Tolerance Index

Relative fresh weight of the embryogenic calli was calculated at all stress levels for different stress regimes according to the following formula:

(1) $$\begin{array}{c} \text{RFW}=\frac{\text{F}{\text{W}}_{f}-\text{F}{\text{W}}_{i}}{\text{F}{\text{W}}_{i}}, \end{array}$$

where FW_*i*_ = initial fresh weight and FW_*f*_ = final fresh weight.

Fresh biomass-based tolerance index (TI) of cotton callus culture was calculated according to the following formula:

(2) TI  (%)=Mean biomass in Cd-stressed mediaMean biomass in control media∗100.  

### 2.4. Assays for Oxidative Stress Biomarkers

Malondialdehyde (MDA) contents were determined according to Zhou and Leul [25]. Briefly, 0.5 g of the cotton calli was homogenized in 10 mL of 0.25% TBA dissolved in 10% trichloroacetic acid (TCA). Homogenate was heated at 95°C for 30 min and then immediately cooled on ice. Later on, it was centrifuged at 5000 rpm for 10 min and the absorbance of the supernatant was measured at 532 nm. Nonspecific absorbance at 600 nm was subtracted from that at 532 nm. The level of lipid peroxidation was expressed as *μ*mol g^−1^ fresh weight by using an extinction coefficient of 155 mM cm^−1^.

In order to determine soluble proteins and various antioxidant enzymes, 0.5 gm of cotton calli was homogenized with a prechilled mortar and pestle under chilled conditions in the extraction buffers specific for each assay. The homogenate was filtered through four layers of muslin cloth. The filtrate was centrifuged at 10000 ×g for 20 min at 4°C and the supernatants were used for various enzymatic assays. Soluble protein contents were determined according to Bradford method [26] using bovine serum albumin as standard.

Superoxide dismutase (SOD) (EC 1.15.1.1) activity was determined based on the method of Zhou et al. [27] by using the photochemical NBT method. The samples (0.5 g) of cotton callus culture were homogenized in 5 mL extraction buffer consisting of 50 mM phosphate, pH 7.8. The assay mixture in 3 mL contained 50 mM phosphate buffer, pH 7.8, 26 mM L-methionine, 750 *μ*M NBT, 1 *μ*M EDTA, and 20 *μ*M riboflavin. The photoreduction of NBT (formation of purple formazan) was measured at 560 nm and an inhibition curve was made against different volumes of extract. One unit of SOD is defined as being present in the volume of extract that causes inhibition of the photoreduction of NBT by 50%.

Hydrogen peroxide (H_2_O_2_) content was determined colorimetrically as described by Jana and Choudhuri [28]. H_2_O_2_ was extracted by homogenizing 0.5 g leaf tissue with 3 mL phosphate buffer (50 mM, pH 6.5). The homogenate was centrifuged at 6000 rpm for 25 min. To determine H_2_O_2_ level, 3 mL of extracted solution was mixed with 1 mL of 0.1% titanium sulfate in 20% H_2_SO_4_. The mixture was then centrifuged at 6000 g for 15 min. The intensity of the yellow color of the supernatant at 410 nm was measured. H_2_O_2_ level was calculated using the extinction coefficient (*E* = 0.28 *μ*M cm^−1^).

Peroxidase (POD) (E.C. 1.11.1.7) activity was measured as described by Zhou and Leul [29] using guaiacol as the substrate in a total volume of 3 mL. The reaction mixture consisted of 50 mM potassium phosphate buffer (pH 6.1), 1% guaiacol, 0.4% H_2_O_2_, and enzyme extract. Increase in the absorbance due to oxidation of guaiacol was measured at 470 nm. Enzyme activity was calculated in terms of absorbance on 470 nm g^−1^ FW per min at 25 ± 2°C.

Assay for ascorbate peroxidase (APX) activity (EC 1.11.1.11.) was carried out according to Nakano and Asada [30] in a reaction mixture in 3 mL containing 100 mM phosphate (pH 7.0), 0.1 mM EDTA-Na_2_, 0.3 mM ascorbic acid, 0.06 mM H_2_O_2_, and 100 *μ*L enzyme extract. The change in absorption at 290 nm was recorded 30 s after the addition of H_2_O_2_. Enzyme activity was quantified using the molar extinction coefficient for ascorbate (*E* = 2.8 mM^−1^ cm^−1^) expressed as *μ*M g^−1^FW.

Catalase (CAT) (EC 1.11.1.6) activity was measured according to Radwan et al.[31]. Briefly, the disappearance of H_2_O_2_ was monitored by measuring the decrease in absorbance at 240 nm (*E* = 0.036 mM^−1 ^cm^−1^) of a reaction mixture consisting of 25 mM potassium phosphate buffer (pH 7.0), 10 mM H_2_O_2_, and enzyme extract. The final activity was expressed as U g^−1^ FW.

### 2.5. Statistical Analyses

The data were subjected to one-way analysis of variance (ANOVA) using SAS (Version 9) software for statistical significance at *P* < 0.05. All the results were the mean ± SE of three replications. Means were separated by least significant difference (LSD) test at 5% level of significance.

## 3. Results and Discussion

Cadmium-induced overproduction of reactive oxygen species (ROS) may cause oxidative damage in plants. To abate such damage, plants develop a complex antioxidant enzymes system [32]. In our present* in vitro *experiment, we studied the physiological and biochemical response reactions of the cotton callus culture under increasing concentrations of Cd for different stress periods.

### 3.1. Growth of Cotton Callus Culture

Growth inhibition in terms of biomass reduction is the initial response of plants to metal toxicity [2]. In order to study the dose-dependent effect of Cd on cotton callus growth under different stress periods, the mean values of relative fresh weight growth rate of cotton callus culture were analyzed (Table 1). At the end of all four stress periods, mean growth values of cotton calli at the highest Cd level (i.e., 1000 *μ*M) were lower than its respective control, while at other three Cd stress levels their mean growth rates were either relatively higher or lower than their relative controls. However, the mean values at 1000 *μ*M Cd levels in different stress periods were statistically nonsignificant compared to the control. Moreover, lower Cd stress levels (550 and 700 *μ*M) caused progressive enhancement in the growth responses of the calli after different stress periods except after 14-day stress duration. Moreover, the highest relative increase (30.53%) over the relevant control was found at 850 *μ*M Cd stress after 28-day stress regime.

In the present* in vitro *experiment, relative fresh weight growth rate was stimulated by application of low level of Cd, while it was decreased at the highest Cd level (1000 *μ*M). Similar to our findings, Gomes-Junior et al. [33] in coffee suspension cells, Fornazier et al. [34] in* Saccharum officinarum* callus cultures, Sobkowiak and Deckert [35] in* G. max*, Hirt et al. [36] in tobacco, and Chakravarty and Srivastava [37] in peanut also found that lower level of Cd stimulated the growth of cell cultures. However, Shekhawat et al. [2] showed reduction in the fresh growth with the increasing concentration of Cd in calli of* Brassica*.

Nevertheless, enhanced growth rate was observed in the first 7-day and last 21- and 28-day stress regimes. The duration dependent (i.e., 7, 21, and 28 days) stimulation in the growth of cotton callus cultures might be due to several reasons. This might be due to the fact that callus cultures utilized the available energy resources of MS medium in the first stress regime. And the overall growth rate was reduced in the second stress phase (14-day period) due to the depletion of energy resources. However, the calli became capable of activating their own genetic potential to make food for themselves in the third and fourth phase of their growth period. Other possible reasons could be that Cd and Zinc (Zn) have almost similar structural, geochemical, and environmental properties and can functionally substitute for Zn in the cell [35]. The stimulatory effect of low Cd concentration on the growth of cells in culture could be explained by competition between Zn and Cd for the same cellular binding sites [35].

### 3.2. Percent Tolerance Index of Cotton Callus Culture

Fresh biomass-based tolerance capability of the cotton callus culture under various Cd stress levels for different duration was also very interesting (Table 2). As tolerance indices were determined using growth responses, so similar trend was observed except at 850 *μ*M Cd level after 21-day stress period. The highest increasing trend was found after 7-day stress regime having highest relative increase (50.35%) over the control among all the stress regimes. At low Cd concentration, callus culture was more tolerant. However, at higher Cd concentration (1000 *μ*M), it was less tolerant. Similar behavior regarding the tolerance index was also observed by Shekhawat et al. [2] with increase in the concentration of Cd.

### 3.3. Soluble Protein Contents of Cotton Callus Culture

Table 3 illustrates the total soluble protein contents in the cotton callus culture grown for four different stress periods using various Cd stress levels. As compared to their respective controls, the cell protein contents of YZ1 tended to decrease after 7-day and 14-day stress treatment except at 850 *μ*M Cd level after 7-day stress period. However, these protein contents showed an inclination over their related controls in later stress periods (i.e., 21 and 28 days). Furthermore, statistically significant and highest decrease was observed at 550 *μ*M Cd after 7-day stress regime, while such trend was found at 700 *μ*M Cd after 14-day stress period. Highest and statistically significant enhancement in the protein contents of the callus culture was found, respectively, at 850 and 1000 *μ*M Cd after 21- and 28-day stress duration. There could be several reasons in this regard. For example, (1) under stressful conditions, plants can synthesize new proteins [38] that might also be in our experiment; (2) furthermore, increase in soluble protein contents in the latter two stress periods reveals that stress-shocked proteins might have been produced in order to combat the Cd-induced stress in cotton callus culture.

### 3.4. MDA Contents of Cotton Callus Culture

Lipid peroxidation in terms of MDA contents is a good indicator of oxidative damage to membranes [39]. In order to quantify the toxic effect of Cd on cell membrane integrity, MDA contents of cotton cell culture were determined (Table 4). As compared to their respective controls, the MDA contents of the stress-shocked callus culture showed quite interesting responses towards various Cd stress levels for all stress periods. For example, among the applied stress conditions, at 550 *μ*M the MDA contents decreased after various stress periods except 21-day stress duration. With the enhancement of Cd levels (700, 850 *μ*M), the mean values of MDA contents increased over the respective controls after all stress applied periods. However, at 1000 *μ*M increasing trend was only observed after 7- and 21-day stress duration.

Enhancement in the MDA contents means that lipid peroxidation was relatively efficient conveying the message that ROS might have been produced. Furthermore, the mean data of the MDA contents reveal that overall increase was not appreciable under different Cd levels. Thus it suggests sufficient ROS detoxification has taken place as evident by an increase in activity of the antioxidative machinery. Our findings are in the line of findings of Gomes-Junior et al. [33] in coffee cells, and Shekhawat et al. [2] in* Brassica* as well as those of Cho and Seo [40] in* Arabidopsis* and Hassan et al. [41] in rice.

### 3.5. SOD Activity of Cotton Callus Culture

SOD is responsible for dismutating superoxide into H_2_O_2_ and thus presents first line of defense against ROS [42]. Increase in SOD activity can be due to increase in ROS [2]. In our present study, we also quantitatively determined the superoxide dismutase (SOD) activity in the cotton callus culture exposed to exceeding Cd stress levels (Table 5). The tabulated data revealed that its activity at 1000 *μ*M Cd improved relative to their respective controls only after 7-, 14-, and 21-day stress periods. This inclination trend could also be obtained at all other applied Cd levels except at 700 and 850 *μ*M Cd after 14-day stress period and 550 and 700 *μ*M Cd after 21-day stress duration, where the mean values of the SOD were lower than the related control. However, the mean values of SOD in the callus culture under various stress levels of Cd after the 28-day stress regime were declined in comparison with the control. Moreover, the SOD activities were almost higher throughout the experimental course, albeit nonsignificant to the relative control except at 700 *μ*M Cd of the 21-day stress experiment. Such enhancement in SOD activity under Cd stress has also been previously reported in different plants species [33, 41, 43–47].

The overall increase in the SOD activities reveals that Cd caused increase in ROS production. However, decrease in its activity in the last two stress periods particularly after 28-day period reveals that calli became capable of bearing the Cd stress. This finding is further supported by a decrease in production of H_2_O_2_ conveying the message that less ROS might have been produced in the later course of Cd stress periods.

### 3.6. H_2_O_2_ Activity of Cotton Callus Culture

H_2_O_2_ plays a dual role in plants: at low concentrations, it acts as a signal molecule in response to various biotic and abiotic stresses and, at high concentrations, it leads to programmed cell death [42].  Table 6 shows mean values of hydrogen peroxide (H_2_O_2_) activity in the upland cotton callus culture grown for various stress durations of Cd stress. According to mean data, there was almost a decreasing trend in the H_2_O_2_ activity in stress-shocked calli over their relevant controls after different stress periods with few exceptions. For example, at 850 *μ*M Cd level the mean values of the activity increased after 7- and 21-day stress period, while this increasing trend was found in the calli at 550 and 700 *μ*M Cd after 14-day stress duration.

According to data, H_2_O_2_ was produced, which signals that Cd stress caused oxidative stress. However, the trend was decreasing one in all stress periods over their respective controls. This decreasing trend might be the key point that the callus culture growth was not affected as a whole. The other fact is that there is an increase in the activity of CAT and in most cases in that of APX. And hence the H_2_O_2_ production is decreasing. All these events signal that ROS scavenging enzymes were active in combating the Cd stress. This shows that the cotton callus culture is resistant to Cd high doses.

### 3.7. POD Activity of Cotton Callus Culture

Peroxidase (POD) is not only one of the defense proteins, but also an important antioxidant enzyme involved in the response to environmental stresses [48]. Cd-imposed stress treatments have shown that POD had transient behavior, which is either increased or decreased in plants [12, 49]. The peroxidase activity (POD) of the cotton callus culture (YZ1) is shown in Table 7. It reveals that, in comparison to respective controls, there was a decreasing behavior of the activity at all Cd levels after different stress periods with few exceptions. For example, after 14-day stress the mean values of the POD activity progressively increased over the control while after 21-day stress regime increase in the activity was only observed at 1000 *μ*M Cd level. Moreover, among all the stress levels as well as stress durations, the highest statistically significant increase (77.45%) over the related control was at 1000 *μ*M Cd after 14-day stress duration while the lowest statistically significant decrease (24.25%) was also at 1000 *μ*M Cd after 7-day stress period.

It shows that the decomposition of H_2_O_2_ at this stage might have been performed by POD. Our present results are not in line with those of Cho and Seo [40] and Gomes-Junior et al. [33].

### 3.8. APX Activity of Cotton Callus Culture

APX is most essential antioxidant enzymes in scavenging ROS due to having higher affinity for H_2_O_2_ (even in *μ*M range) [42]. It can detoxify H_2_O_2_ under abiotic stress conditions [50]. The ascorbate peroxidase (APX) activity also greatly varied in the experimental cotton callus culture in our present study (Table 8). Its activity descended over their relevant controls in the first three stress regimes except at 700 *μ*M Cd after 14- and 21-day stress duration. However, after 28-day stress regimes, the APX activity first decreased at 550 *μ*M Cd and incrementally enhanced at all other Cd stress levels. The data further reveals that the observed decrease was statistically significant only at 1000 *μ*M Cd after 14-day stress shocks. And the significant relative was only found at 850 *μ*M Cd after the 28-day stress period.

This decreasing behavior was also noticed by Shekhawat et al. [2] during their studies in callus cultures of* Brassica*. The overall inactivation of APX enzyme might be due to metal-sulfhydryl binding Shekhawat et al. [2]. Our results are consistent with findings of Sharma et al. [51] in barley and Israr et al. [52] in* Sesbania* callus.

### 3.9. CAT Activity of Cotton Callus Culture

CAT is among the H_2_O_2_-scavenging enzymes. The balance between the activity of H_2_O_2_-producing and H_2_O_2_-scavenging enzymes plays an important role in providing a plant defense mechanism against any oxidative damage [38]. The CAT activity also showed obvious results in cotton callus culture after various Cd stressful regimes (Table 9). In comparison to their related controls, its mean values significantly increased after 7-, 21-, and 28-day Cd treatment. However, there was found a decreasing trend over the control with the addition of more Cd in the growing medium after 14-day stress time except at 1000 *μ*M Cd, where the CAT activity was 63.55% higher over its respective control. Moreover, after 7-day stress period, the CAT activity linearly increased, while in case of 21- and 28-day stress regimes the increase in the activity over the respective control was severalfold higher with increase in the concentration of Cd.

In our present study, the CAT activity was activated as a whole. That is why the overall fresh biomass production showed upward trend. So it means that POD was active to decompose H_2_O_2_ produced as a result of SOD activity. Similar upward trends have been noticed by other workers [2, 33, 34, 43, 44, 49]. However, there was observed a decrease in the CAT activity of* Brassica *callus by Shekhawat et al. [2] and Sandalio et al. [53] in* Pisum sativum.*

Both APX and CAT showed dissimilar trend in our present study. This might be because both enzymes are working on the same substrate (H_2_O_2_). Therefore, the detoxification of H_2_O_2_ occurred mainly through CAT and that is why APX activity was declined due to the lesser availability of substrate. Another possible reason for the decreased APX activity could be induced inactivation of APX enzyme.

## 4. Conclusion

- Cell growth and MDA contents are the two important indicators which show whether oxidative damage has been caused or not. Here in case of our present study cell growth in terms of relative fresh weight growth rates was not significantly affected. However, the lipid peroxidation was relatively efficient conveying the message that ROS have been produced. This is further testified by the increase in the activity of SOD, CAT, and so forth.
- In our present findings, the overall H_2_O_2_ activity was downregulated in all stress periods over their respective controls. So due to less production of H_2_O_2_, the overall growth efficiency of the callus under Cd-exposed conditions was unaffected.
- The high MDA contents and SOD show that membrane damage and oxidative stress have been caused. However, low H_2_O_2_ concentrations establish that this is presumably suppressed by the strong antioxidant system prevailing in cotton callus culture more importantly in the order of CAT > APX > POD.
- The present study set a new avenue to explore the molecular mechanisms in cotton callus culture both at genetic and proteomic levels under Cd stress.

## Figures and Tables

**Table 1:** Relative fresh weight of cotton callus culture (cv. YZ1) grown under various levels of cadmium.

Cd treatment (µM)	Stress periods			
	7 days	14 days	21 days	28 days
0	0.33 ± 0.01^a^ (0.00)	1.87 ± 0.45^a^ (0.00)	2.96 ± 0.48^a^ (0.00)	2.59 ± 0.17^ab^ (0.00)
550	0.36 ± 0.02^a^ (−7.23)	1.88 ± 0.49^a^ (−0.39)	3.50 ± 0.27^a^ (−18.11)	2.83 ± 0.63^ab^ (−9.32)
700	0.45 ± 0.05^a^ (−34.18)	1.51 ± 0.15^ab^ (19.21)	3.06 ± 0.27^a^ (−3.39)	2.74 ± 0.27^ab^ (−5.58)
850	0.49 ± 0.09^a^ (−47.54)	1.49 ± 0.15^ab^ (20.37)	2.91 ± 0.46^a^ (1.81)	3.38 ± 0.17^a^ (−30.53)
1000	0.30 ± 0.09^a^ (8.72)	0.69 ± 0.12^b^ (62.93)	2.59 ± 0.21^a^ (12.48)	2.32 ± 0.14^b^ (10.37)

Values are the means ± SE of three replications. Variants possessing the same letters are not statistically significant at 5% probability level. Values in parenthesis are relative inhibition (% relative inhibition = 1 − mean values in treatment/mean values in respective control ∗ 100). −ve values show increase and +ve values show decrease over the related controls.

**Table 2:** Percent tolerance index of cotton callus culture (cv. YZ1) grown under various levels of cadmium.

Cd treatment (µM)	Stress periods			
	7 days	14 days	21 days	28 days
0	100.00 ± 0.00^a^ (0.00)	100.00 ± 0.00^a^ (0.00)	100.00 ± 0.00^a^ (0.00)	100.00 ± 0.00^b^ (0.00)
550	107.97 ± 9.25^a^ (−7.97)	102.64 ± 18.08^a^ (−2.65)	122.19 ± 13.13^a^ (−22.19)	106.93 ± 18.69^ab^ (−6.93)
700	133.41 ± 8.69^a^ (−33.41)	92.17 ± 23.62^a^ (7.83)	106.73 ± 11.22^a^ (−6.73)	105.5 ± 6.26^ab^ (−5.50)
850	150.35 ± 32.02^a^ (−50.35)	87.07 ± 18.87^ab^ (12.93)	102.63 ± 19.94^a^ (−2.63)	131.41 ± 8.62^ab^ (−31.41)
1000	91.79 ± 29.21^a^ (8.21)	38.40 ± 3.97^b^ (61.60)	90.45 ± 9.46^a^ (9.55)	90.00 ± 4.69^b^ (10.00)

Values are the means ± SE of three replications. Variants possessing same letters are not statistically significant at 5% probability level. Other pieces of information are the same as in Table 1.

**Table 3:** Soluble protein (mgg^−1^ FW) of cotton callus culture (cv. YZ1) grown under various levels of cadmium.

Cd treatment (µM)	Stress periods			
	7 days	14 days	21 days	28 days
0	4.08 ± 0.07^ab^ (0.00)	6.53 ± 0.17^a^ (0.00)	6.92 ± 0.21^d^ (0.00)	4.99 ± 0.19^d^ (0.00)
550	2.19 ± 0.21^c^ (46.44)	4.77 ± 0.19^b^ (27.04)	7.78 ± 0.21^c^ (−12.53)	6.88 ± 0.15^b^ (−37.90)
700	3.82 ± 0.19^ab^ (6.52)	2.89 ± 0.14^d^ (55.87)	7.82 ± 0.16^c^ (−13.01)	5.89 ± 0.14^c^ (−18.18)
850	4.30 ± 0.11^a^ (−5.40)	3.82 ± 0.17^c^ (41.53)	14.87 ± 0.18^a^ (−114.94)	7.21 ± 0.15^b^ (−44.59)
1000	3.60 ± 0.29^b^ (11.66)	4.59 ± 0.19^b^ (29.64)	8.85 ± 0.18^b^ (−28.00)	9.33 ± 0.14^a^ (−87.17)

Values are the means ± SE of three replications. Variants possessing the same letters are not statistically significant at 5% probability level. Other pieces of information are the same as in Table 1.

**Table 4:** MDA contents (nmolg^−1^FW) of cotton callus culture (cv. YZ1) grown under various levels of cadmium.

Cd treatment (µM)	Stress periods			
	7 days	14 days	21 days	28 days
0	5.83 ± 0.42^bc^ (0.00)	9.16 ± 0.85^a^ (0.00)	6.56 ± 1.15^b^ (0.00)	11.35 ± 0.00^ab^ (0.00)
550	5.24 ± 0.58^c^ (10.04)	7.05 ± 0.00^b^ (23.03)	10.49 ± 1.00^a^ (−59.95)	9.81 ± 0.70^b^ (13.64)
700	8.09 ± 0.00^a^ (−38.72)	9.63 ± 0.30^a^ (−5.12)	8.60 ± 1.39^ab^ (−31.11)	12.73 ± 0.20^ab^ (−12.12)
850	8.60 ± 0.20^a^ (−47.57)	9.46 ± 0.40^a^ (−3.25)	10.32 ± 1.19^a^ (−57.33)	13.96 ± 1.55^a^ (−22.99)
1000	6.79 ± 0.43^b^ (−16.54)	9.12 ± 0.10^a^ (0.51)	9.29 ± 0.10^ab^ (−41.60)	10.32 ± 1.29^b^ (9.09)

Values are the means ± SE of three replications. Variants possessing the same letters are not statistically significant at 5% probability level. Other pieces of information are the same as in Table 1.

**Table 5:** SOD activity (Ug^−1^FW) of cotton callus culture (cv. YZ1) grown under various levels of cadmium.

Cd treatment (µM)	Stress periods			
	7 days	14 days	21 days	28 days
0	319.51 ± 25.74^a^ (0.00)	360.76 ± 40.14^a^ (0.00)	433.14 ± 70.17^ab^ (0.00)	372.77 ± 9.67^a^ (0.00)
550	326.98 ± 54.95^a^ (−2.34)	414.35 ± 25.90^a^ (−14.85)	305.76 ± 12.09^bc^ (29.41)	239.03 ± 55.04^b^ (35.88)
700	406.67 ± 7.47^a^ (−27.22)	332.49 ± 39.79^a^ (7.84)	230.37 ± 24.18^c^ (46.81)	230.37 ± 12.09^b^ (38.20)
850	358.63 ± 64.60^a^ (−12.24)	350.64 ± 25.52^a^ (2.81)	443.58 ± 74.44^ab^ (−2.41)	322.51 ± 7.25^ab^ (13.48)
1000	372.99 ± 21.72^a^ (−16.74)	393.36 ± 38.98^a^ (−9.03)	509.30 ± 44.76^a^ (−17.58)	371.59 ± 67.72^a^ (0.32)

Values are the means ± SE of three replications. Variants possessing the same letters are not statistically significant at 5% probability level. Other pieces of information are the same as in Table 1.

**Table 6:** H_2_O_2_ (*μ*M g^−1^FW) of cotton callus culture (cv. YZ1) grown under various levels of cadmium.

Cd treatment (µM)	Stress periods			
	7 days	14 days	21 days	28 days
0	71.86 ± 10.29^a^ (0.00)	58.94 ± 6.46^bc^ (0.00)	38.15 ± 2.55^ab^ (0.00)	45.98 ± 6.20^a^ (0.00)
550	45.88 ± 5.69^b^ (36.16)	77.97 ± 7.95^ab^ (−32.28)	35.16 ± 2.39^b^ (7.84)	36.71 ± 5.43^a^ (20.18)
700	58.87 ± 3.75^ab^ (18.08)	81.71 ± 7.45^a^ (−38.63)	36.85 ± 5.10^ab^ (3.40)	37.26 ± 5.40^a^ (18.98)
850	75.93 ± 6.89^a^ (−5.66)	36.79 ± 5.72^d^ (37.58)	46.31 ± 3.21^a^ (−21.40)	39.76 ± 3.43^a^ (13.54)
1000	65.34 ± 3.89^ab^ (9.07)	49.97 ± 3.32^cd^ (15.21)	30.43 ± 2.71^b^ (20.23)	40.60 ± 6.84^a^ (11.72)

Values are the means ± SE of three replications. Variants possessing the same letters are not statistically significant at 5% probability level. Other pieces of information are the same as in Table 1.

**Table 7:** POD activity (OD470 g^−1^FW/min) of cotton callus culture (cv. YZ1) grown under various levels of cadmium.

Cd treatment (µM)	Stress periods			
	7 days	14 days	21 days	28 days
0	75.96 ± 5.10^a^ (0.00)	52.77 ± 1.98^c^ (0.00)	15.50 ± 0.88^a^ (0.00)	17.63 ± 1.07^a^ (0.00)
550	54.47 ± 1.94^b^ (28.30)	57.80 ± 3.77^cd^ (−9.54)	14.13 ± 0.29^a^ (8.82)	13.37 ± 0.04^c^ (24.20)
700	60.63 ± 1.31^b^ (20.18)	61.72 ± 0.22^c^ (−16.96)	14.27 ± 0.21^a^ (7.96)	15.10 ± 0.04^b^ (14.37)
850	66.44 ± 5.31^ab^ (12.54)	79.73 ± 2.71^b^ (−51.11)	14.53 ± 0.42^a^ (6.24)	16.13 ± 0.12^ab^ (8.51)
1000	57.54 ± 6.97^b^ (24.25)	93.63 ± 3.50^a^ (−77.45)	16.10 ± 1.40^a^ (−3.87)	14.63 ± 0.10^bc^ (17.02)

Values are the means ± SE of three replications. Variants possessing the same letters are not statistically significant at 5% probability level. Other pieces of information are the same as in Table 1.

**Table 8:** APX activity (µMg^−1^FW) of cotton callus culture (cv. YZ1) grown under various levels of cadmium.

Cd treatment (µM)	Stress periods			
	7 days	14 days	21 days	28 days
0	18.16 ± 1.42^a^ (0.00)	12.74 ± 1.13^b^ (0.00)	48.21 ± 1.49^a^ (0.00)	49.49 ± 1.94^bc^ (0.00)
550	9.72 ± 1.09^c^ (46.44)	8.14 ± 0.62^c^ (36.11)	48.00 ± 0.62^a^ (0.44)	47.59 ± 1.75^c^ (3.85)
700	12.64 ± 1.82^bc^ (30.39)	17.06 ± 1.61^a^ (−33.87)	49.50 ± 1.45^a^ (−2.67)	50.36 ± 0.49^bc^ (−1.75)
850	16.76 ± 0.85^ab^ (7.70)	10.07 ±0.50^bc^ (20.98)	47.14 ± 3.34^a^ (2.22)	58.76 ± 2.01^a^ (−18.72)
1000	13.29 ± 2.60^abc^ (26.84)	3.85 ± 0.64^d^ (67.32)	47.80 ± 1.44^a^ (0.85)	53.41 ± 0.92^b^ (−6.51)

Values are the means ± SE of three replications. Variants possessing the same letters are not statistically significant at 5% probability level. Other pieces of information are the same as in Table 1.

**Table 9:** CAT activity (U/gFW) of cotton callus culture (cv. YZ1) grown under various levels of cadmium.

Cd treatment (µM)	Stress periods			
	7 days	14 days	21 days	28 days
0	2.88 ± 0.24^d^ (0.00)	5.71 ± 0.14^b^ (0.00)	4.57 ± 0.24^c^ (0.00)	16.573 ± 22.30^a^ (0.00)
550	4.76 ± 0.21^b^ (−65.47)	3.24 ± 0.42^c^ (43.22)	38.84 ± 5.37^b^ (−749.82)	22.95 ± 5.66^c^ (−38.48)
700	3.62 ± 0.25^c^ (−25.72)	3.62 ± 0.27^c^ (36.57)	79.17 ± 11.42^a^ (−1632.46)	56.99 ± 5.37^bc^ (−243.89)
850	4.95 ± 0.12^b^ (−72.07)	2.28 ± 0.16^d^ (59.99)	71.72 ± 15.87^a^ (−1469.44)	44.77 ± 6.27^c^ (−170.11)
1000	6.66 ± 0.24^a^ (−131.40)	9.33 ± 0.17^a^ (−63.55)	12.20 ± 2.54^bc^ (−167.03)	88.88 ± 9.45^b^ (−436.28)

Values are the means ± SE of three replications. Variants possessing the same letters are not statistically significant at 5% probability level. Other pieces of information are the same as in Table 1.

## Abbreviations

APX: Ascorbate peroxidase
CAT: Catalase
MDA: Malondialdehyde
POD: Peroxidase
ROS: Reactive oxygen species
SOD: Superoxide dismutase
H_2_O_2_: Hydrogen peroxide.

## References

[1] Daud MK, Sun YQ, Dawood M (2009). Cadmium-induced functional and ultrastructural alterations in roots of two transgenic cotton cultivars. *Journal of Hazardous Materials*.

[2] Shekhawat GS, Verma K, Jana S, Singh K, Teotia P, Prasad A (2010). *In vitro* biochemical evaluation of cadmium tolerance mechanism in callus and seedlings of *Brassica juncea*. *Protoplasma*.

[3] Prasad MNV (1995). Cadmium toxicity and tolerance in vascular plants. *Environmental and Experimental Botany*.

[4] Foyer C, Lopez-Delgado H, Dat JF, Scott I (1997). Hydrogen peroxide- and glutathione-associated mechanisms of acclimatory stress tolerance and signalling. *Physiologia Plantarum*.

[5] Olmos E, Martínez-Solano JR, Piqueras A, Hellín E (2003). Early steps in the oxidative burst induced by cadmium in cultured tobacco cells (BY-2 line). *Journal of Experimental Botany*.

[6] Zhang H, Jiang Y, He Z, Ma M (2005). Cadmium accumulation and oxidative burst in garlic (*Allium sativum*). *Journal of Plant Physiology*.

[7] Shekhawat GS, Prasad A, Verma K (2008). Changes in growth, lipid peroxidation and antioxidant system in seedlings of *Brassica juncea* (L.) czern. under cadmium stress. *Biochemical and Cellular Archives*.

[8] Ouariti O, Gouia H, Ghorbal MH (1997). Responses of bean and tomato plants to cadmium: growth, mineral nutrition, and nitrate reduction. *Plant Physiology and Biochemistry*.

[9] Molina AS, Nievas C, Chaca MVP (2008). Cadmium-induced oxidative damage and antioxidative defense mechanisms in *Vigna mungo* L. *Plant Growth Regulation*.

[10] Verma K, Shekhawat GS, Sharma A, Mehta SK, Sharma V (2008). Cadmium induced oxidative stress and changes in soluble and ionically bound cell wall peroxidase activities in roots of seedling and 3-4 leaf stage plants of *Brassica juncea* (L.) czern. *Plant Cell Reports*.

[11] Fodor A, Szabo-Nagy A, Erdei L (1995). The effects of cadmium on the fluidity and H^+^-ATPase activity of plasma membrane from sunflower and wheat roots. *Journal of Plant Physiology*.

[12] Meloni DA, Oliva MA, Martinez CA, Cambraia J (2003). Photosynthesis and activity of superoxide dismutase, peroxidase and glutathione reductase in cotton under salt stress. *Environmental and Experimental Botany*.

[13] Niknam V, Meratan AA, Ghaffari SM (2011). The effect of salt stress on lipid peroxidation and antioxidative enzymes in callus of two *Acanthophyllum* species. *In Vitro Cellular & Developmental Biology: Plant*.

[14] Somashekaraiah BV, Padmaja K, Prassad ARK (1992). Phytotoxicity of cadmium ions on germinating seedlings of mung bean (*Phaseolus vulgaris*): involvement of lipid peroxides in chlorophyll degradation. *Physiologia Plantarum*.

[15] Chaoui A, Mazhoudi S, Ghorbal MH, El Ferjani E (1997). Cadmium and zinc induction of lipid peroxidation and effects on antioxidant enzyme activities in bean (*Phaseolus vulgaris* L.). *Plant Science*.

[16] Shekhawat GS, Batra A, Mathur S (2002). A reliable *in vitro* protocol for rapid mass propagation of *Azadirachta indica* Juss. *Journal of Plant Biology*.

[17] Mathur S, Batra A, Shekhawat GS (2002). An efficient *in vitro* method for mass propagation of *Salvadora persica* via apical meristem. *Journal of Plant Biochemistry and Biotechnology*.

[18] Mathur S, Shekhawat GS, Batra A (2002). Micropropagation of *Salvadora persica* Linn. via cotyledonary nodes. *Indian Journal of Biotechnology*.

[19] Mathur S, Shekhawat GS, Batra A (2008). Somatic embryogenesis and plantlet regeneration from cotyledon explants of *Salvadora persica* L. *Phytomorphology*.

[20] Gallego SM, Benavides MP, Tomaro ML (1996). Effect of heavy metal ion excess on sunflower leaves; evidence for involvement of oxidative stress. *Plant Science*.

[21] Sobkowiak R, Rymer K, Rucinska R, Deckert J (2004). Cadmium-induced changes in antioxidant enzymes in suspension culture of soybean cells. *Acta Biochimica Polonica*.

[22] Gossett DR, Millhollon EP, Lucas MC, Banks SW, Marney MM (1994). The effects of NaCl on antioxidant enzyme activities in callus tissue of salt-tolerant and salt-sensitive cotton cultivars (*Gossypium hirsutum* L.). *Plant Cell Reports*.

[23] Garratt LC, Janagoudar BS, Lowe KC, Anthony P, Power JB, Davey MR (2002). Salinity tolerance and antioxidant status in cotton cultures. *Free Radical Biology and Medicine*.

[24] Murashige T, Skoog F (1962). A revised medium for rapid growth and bioassays with tobacco tissue cultures. *Physiolgia Plantarum*.

[25] Zhou WJ, Leul M (1998). Uniconazole-induced alleviation of freezing injury in relation to changes in hormonal balance, enzyme activities and lipid peroxidation in winter rape. *Plant Growth Regulation*.

[26] Bradford MM (1976). A rapid and sensitive method for the quantitation of microgram quantities of protein utilizing the principle of protein dye binding. *Analytical Biochemistry*.

[27] Zhou W, Zhao D, Lin X (1997). Effects of waterlogging on nitrogen accumulation and alleviation of waterlogging damage by application of nitrogen fertilizer and mixtalol in winter rape (*Brassica napus* L.). *Journal of Plant Growth Regulation*.

[28] Jana S, Choudhuri MA (1981). Glycolate metabolism of three submersed aquatic angiosperms during ageing. *Aquatic Botany*.

[29] Zhou WJ, Leul M (1999). Uniconazole-induced tolerance of rape plants to heat stress in relation to changes in hormonal levels, enzyme activities and lipid peroxidation. *Plant Growth Regulation*.

[30] Nakano Y, Asada K (1981). Hydrogen peroxide is scavenged by ascorbate-specific peroxidase in spinach chloroplasts. *Plant and Cell Physiology*.

[31] Radwan DEM, Fayez KA, Mahmoud SY, Hamad A, Lu G (2006). Salicylic acid alleviates growth inhibition and oxidative stress caused by zucchini yellow mosaic virus infection in *Cucurbita pepo* leaves. *Physiological and Molecular Plant Pathology*.

[32] Joseph B, Jini D (2011). Development of salt stress-tolerant plants by gene manipulation of antioxidant enzymes. *Asian Journal of Agricultural Research*.

[33] Gomes-Junior RR, Moldes CA, Delite FS (2006). Antioxidant metabolism of coffee cell suspension cultures in response to cadmium. *Chemosphere*.

[34] Fornazier RF, Ferreira RR, Pereira GJG (2002). Cadmium stress in sugar cane callus cultures: effect on antioxidant enzymes. *Plant Cell, Tissue and Organ Culture*.

[35] Sobkowiak R, Deckert J (2003). Cadmium-induced changes in growth and cell cycle gene expression in suspension-culture cells of soybean. *Plant Physiology and Biochemistry*.

[36] Hirt H, Casari G, Barta A (1989). Cadmium-enhanced gene expression in suspension-culture cells of tobacco. *Planta*.

[37] Chakravarty B, Srivastava S (1992). Toxicity of some heavy metals *in vivo* and *in vitro* in *Helianthus annuus*. *Mutation Research*.

[38] Torabi S, Niknam V (2011). Effects of iso-osmotic concentrations of NaCl and mannitol on some metabolic activity in calluses of two *Salicornia* species. *In Vitro Cellular & Developmental Biology: Plant*.

[39] Dacosta M, Huang B (2007). Changes in antioxidant enzyme activities and lipid peroxidation for bentgrass species in response to drought stress. *Journal of the American Society for Horticultural Science*.

[40] Cho U, Seo N (2005). Oxidative stress in *Arabidopsis thaliana* exposed to cadmium is due to hydrogen peroxide accumulation. *Plant Science*.

[41] Hassan MJ, Zhang GP, Wu FB, Wei K, Chen Z (2005). Zinc alleviates growth inhibition and oxidative stress caused by cadmium in rice. *Journal of Plant Nutrition and Soil Science*.

[42] Gill SS, Tuteja N (2010). Reactive oxygen species and antioxidant machinery in abiotic stress tolerance in crop plants. *Plant Physiology and Biochemistry*.

[43] Vitória AP, Lea PJ, Azevedo RA (2001). Antioxidant enzymes responses to cadmium in radish tissues. *Phytochemistry*.

[44] Wu FB, Zhang G, Dominy P (2003). Four barley genotypes respond differently to cadmium: lipid peroxidation and activities of antioxidant capacity. *Environmental and Experimental Botany*.

[45] Guo TR, Zhang GP, Zhou MX, Wu F, Chen J (2004). Effects of aluminum and cadmium toxicity on growth and antioxidant enzyme activities of two barley genotypes with different Al resistance. *Plant and Soil*.

[46] Hsu YT, Kao CH (2004). Cadmium toxicity is reduced by nitric oxide in rice leaves. *Plant Growth Regulation*.

[47] Benavides MP, Gallego SM, Tomaro ML (2005). Cadmium toxicity in plants. *Brazilian Journal of Plant Physiology*.

[48] Flohé L, Ursini F (2008). Peroxidase: a term of many meanings. *Antioxidants & Redox Signaling*.

[49] Balestrasse KB, Gardey L, Gallego SM, Tomaro ML (2001). Response of antioxidant defence system in soybean nodules and roots subjected to cadmium stress. *Australian Journal of Plant Physiology*.

[50] Gratão PL, Polle A, Lea PJ, Azevedo RA (2005). Making the life of heavy metal-stressed plants a little easier. *Functional Plant Biology*.

[51] Sharma YK, León J, Raskin I, Davis KR (1996). Ozone-induced responses in *Arabidopsis thaliana*: the role of salicylic acid in the accumulation of defense-related transcripts and induced resistance. *Proceedings of the National Academy of Sciences of the United States of America*.

[52] Israr M, Sahi SV, Jain J (2006). Cadmium accumulation and antioxidative responses in the *Sesbania drummondii* callus. *Archives of Environmental Contamination and Toxicology*.

[53] Sandalio LM, Dalurzo HC, Gomez M, Romero-Puertas MC, del Río LA (2001). Cadmium-induced changes in the growth and oxidative metabolism of pea plants. *Journal of Experimental Botany*.

